# The invasion process of bovine erythrocyte by *Babesia divergens*: knowledge from an in vitro assay

**DOI:** 10.1186/1297-9716-42-62

**Published:** 2011-05-11

**Authors:** Yi Sun, Emmanuelle Moreau, Alain Chauvin, Laurence Malandrin

**Affiliations:** 1 ONIRIS, UMR1300, Biologie, Epidémiologie et Analyse de Risque en Santé Animale, Route de Gachet, La Chantrerie, BP 40706, F-44307 Nantes, France; 2 INRA Angers-Nantes, BP 71627, F-44307 Nantes, France; 3 Université Nantes Angers Le Mans, France

## Abstract

*Babesia divergens *is a tick-transmitted apicomplexan parasite for which asexual multiplication in its vertebrate hosts is restricted to erythrocytes. Current knowledge of invasion of these target cells is limited. An efficient in vitro invasion assay was set up to gain access to this information. Parasites prepared from infected RBC, lysed by electroporation, and mixed with bovine RBC in a selected synthetic medium (RPMI 1640 supplemented with calcium) were able to establish subsequent cultures with parasitemia ranging from 6 to 14%. Free parasites remaining in the invasion medium could be eliminated by Percoll gradient and culture could be pursued with the freshly invaded erythrocytes. In this way, the invasion time window could be shortened to obtain a synchronised start of the culture or to study the kinetics of invasion. With this assay we demonstrate that 1) erythrocyte invasion by *B. divergens *is a rapid process since 70% of the invasion-competent parasites invaded the RBC in less than 45 s; 2) all invasion-competent parasites achieved invasion within 10 min of contact; 3) one erythrocyte could be invaded concomitantly by two merozoites; 4) despite a synchronous start, the parasite population evolved heterogeneously resulting in a progressive loss of synchronisation. Western blot analysis of proteins collected from invasion medium were performed with sera from animals experimentally infected with *B. divergens *and highlighted several proteins. The dose-dependent, inhibitory effects of these sera on *B. divergens *invasion suggest that these proteins might be involved in the invasion process. Further investigations are required for their characterisation.

## Introduction

*Babesia divergens *is a tick-transmitted intra-erythrocytic apicomplexan parasite, which infects cattle and a wide variety of other mammals. Experimental or natural infections by *B. divergens *have been documented in gerbils, sheep, and reindeer [1-4]. *B. divergens *has also been recognised, in the last 30 years, as a zoonotic agent in Europe [5-7].

Apicomplexan blood parasites differ in the range of cell types that they infect. *Plasmodium spp*. or *Theileria spp*. sporozoites first invade hepatocytes or lymphocytes, respectively, and then evolve into erythrocyte invasive merozoites [8]. In constrast, the sporozoite and the merozoite of *Babesia spp*., two infectious forms of the parasite, directly invade the host erythrocyte where they multiply asexually [9]. It is obvious that inhibition of their multiplication, and especially the step of invasion, should prevent the disease. Molecules engaged in the invasion process or contributing to its regulation are proposed as promising drug targets. In addition, since both the sporozoite and merozoite of *Babesia *are infectious to RBC, and since identical molecules involved in erythrocyte invasion are expressed in both stages [10,11], these proteins might provide a common target for antibody-mediated inhibition of invasion.

The process of erythrocyte invasion by *B. divergens *is considered to be similar to that of *Plasmodium*. It is described as an initial recognition between the zoite and host cell, immediately followed by progressive internalisation at the site of merozoite apical contact, and eventually the closure of the parasitophorous vacuole [12]. Rapid invasion has been observed for *Plasmodium *and other Apicomplexan parasites. A recent study on *P. falciparum *demonstrated that merozoites recognize new target RBC within 1 min after their release from the host RBC [13]. Eighty percent of the invasion events occured within 10 min of mixing merozoites and RBC [14]. Parasite entry ensued and was complete on average 27.6 s after primary contact [13]. For *P. knowlesi*, erythrocyte invasion was shown to be accomplished within minutes after the initial contact [15]. Moreover, the penetration of *Toxoplasma gondii *into a vacuole formed by invagination of the plasma membrane within 25-40 s was also documented [16]. However, the cellular interactions between *Babesia *and its host cell have not yet been fully described, notably the disappearance of the parasitophorous vacuole, a Piroplasmidae-specific feature.

Characterisation of the molecules involved in the invasion process, as well as their corresponding erythrocyte receptors, is basic information necessary for the comprehension of RBC invasion. The proteins located on the merozoite surface, for example Bd37 of *B. divergens*, are usually involved in RBC adhesion [17] and are shed during the parasite internalisation process [18]. The molecules harboured in the characteristic apicomplexan secretory organelles, rhoptries, micronemes and dense granules (spherical bodies for the genus *Babesia*), are secreted during the invasion process by *Plasmodium*, *Toxoplasma*, and *Eimeria *[19-21] and are generally involved in parasite internalisation [22]. Two of these molecules have been identified in *B. divergens*, AMA-1 (Apical Membrane Antigen-1) and RAP-1 (Rhoptry Associated Protein-1). AMA-1 is secreted from the micronemes and has been characterised in hemoprotozoans in all *Plasmodium *species analysed [23] as well as in *B. bovis *[24] and *B. divergens *[25]. In *Plasmodium*, it is apparently involved in host-cell recognition, binding, and possibly motility, however its interaction with the host erythrocyte remains controversial [26]. RAP-1 has been evidenced in several *Babesia *species [27-30], and in *B. bovis*, it is supposed to be involved in erythrocyte binding and parasitophorous vacuole formation [31]. These parasitic invasion-related antigens are decapped or secreted into the surroundings during the invasion process [32]. It should therefore be possible to characterise them from the invasion medium.

In summary, the aim of our work was to set up an in vitro *B. divergens *invasion assay to characterise this process, to obtain a synchronised culture in order to focus on stage-specific activities of the parasites, and to collect the proteins in the invasion medium for further characterisation in order to elucidate the invasion mechanism.

## Materials and methods

### In vitro culture of *B. divergens*

*B. divergens *Rouen 87 was isolated from a human in 1987, then kept at Oniris (France), and was cultivated in vitro as described previously [33]. Briefly, parasites were cultured in bovine erythrocytes at 5% haematocrit in RPMI 1640 (Lonza, Belgium) supplemented with 20% of heat-inactivated fetal calf serum (FCS, Lonza, Belgium). Cultures were incubated in a humidified 6% CO_2 _atmosphere at 37°C, and amplified in culture flasks to obtain the volume (up to 500 mL) and parasitemia (up to 25%) required for each experiment.

### Methods for preparing free parasites

Three different methods were used to prepare live *B. divergens *parasites. First of all, free merozoites were collected from the culture medium by Percoll gradient separation. Briefly, cultures were centrifuged (2 000 *g*, 5 min) and the pellet was diluted to 1:3 with RPMI 1640. This suspension was then layered on top of a 1.08 g/mL iso-osmotic Percoll solution prepared according to the manufacturer's instructions (Amersham Bioscience, Piscataway, USA) and centrifuged (2 000 *g*, 10 min, 20°C). The merozoites collected at the interface were washed twice in PBS (4 000 *g*, 10 min, 4°C). Besides the collection of free merozoites, the intra-erythrocytic parasites could also be liberated by RBC lysis using mechanical (high-voltage electroporation) or chemical methods (osmotic lysis). The electroporation protocol described for *B. bovis *was tested [32]. Parasitised cultures were centrifuged and the pellet (2 mL) was resuspended in an equal volume of cytomix (120 mM KCl, 0.15 mM CaCl_2_, 2 mM EGTA, 5 mM MgCl_2_, 10 mM K_2_HPO_4_/KH_2_PO_4 _pH 8.0, 25 mM HEPES pH 8.0). Samples of 750 μL were subjected to 5 intermittent (10 s, 0°C) high-voltage pulses (2.5 kV, 200 Ω, 25 μF) in a BioRad (Hercules, USA) Gene Pulser with a pulse controller using 4-mm BioRad cuvettes to lyse RBC. To eliminate the few remaining non-lysed RBC, the liberated parasites were collected by Percoll gradient and washed as described before. Osmotic lysis of the RBC was obtained by mixing a 2 mL pellet of infected RBC with 23 mL cytoyi (cytomix modification: 12.6 mM K_2_HPO_4, _8.74 mM KH_2_PO_4_, pH 6.5) at room temperature for 5 min, and then washing with PBS. The pelleted parasites obtained with the three methods tested, were examined on Giemsa-stained smears, and mixed with the invasion medium to test their viability by in vitro invasion assays.

### In vitro invasion media

A synthetic medium was sought which allowed comparable invasion efficiency to that obtained with the culture medium (RPMI 1640 with 20% FCS). Three synthetic media candidates (RPMI 1640, PBS, Tris-Sucrose) were therefore tested with or without the addition of 1 mM calcium or 1 mM magnesium. The culture medium was used as control.

### In vitro invasion assay and evaluation of invasion efficiency

The invasion medium as well as the culture medium used as control were prepared with 5% RBC in 24-well plates (2 mL/well) and pre-warmed at 37°C. The pelleted parasites collected as previously described were added to each well. The efficiency of the invasion process was determined by counting the infected RBC on Giemsa-stained smears. Percoll separation was used to eliminate the non-invasive or dead parasites located on the surface of RBC. The effect of the Percoll separation was checked by performing an invasion assay using free merozoites collected from culture and incubated up to 120 min in RPMI 1640 with RBC. The viability of the intra-erythrocytic parasites was then controlled by washing cells collected after Percoll separation with RPMI 1640, cultivating them for 5 h, then checking them for parasitemia. Parasitemia was calculated at each step (before, after Percoll separation, and after culture), on at least 2 000 RBC.

### Kinetics of invasion

The kinetics of invasion was followed in the selected synthetic medium (RPMI 1640 + 1 mM CaCl_2_), using parasites prepared by electroporation or osmotic lysis. For this purpose, RBC and parasites were mixed and harvested 0 min, 1.5 min, 3 min, 5 min, 10 min, 20 min, 30 min, 45 min, 60 min, 120 min, 160 min, and 240 min after initial contact. They were either observed directly on Giemsa-stained smears for morphological analysis, or centrifuged with Percoll gradient and reincubated in culture medium for parasitemia counting. Due to the speed of invasion, a rapid gradient Percoll separation was used: A layer of 500 μL medium was placed on top of 500 μL of Percoll in an Eppendorff tube, then centrifuged (30 s, 16 000 *g*) and washed with RPMI 1640. Each pellet was resuspended in 200 μL of culture medium and transferred to a 96-well microplate for further incubation.

### Origin of sera used

Seven immune sera were used to recognise the proteins collected in the supernatant during invasion. One serum was prepared from a rabbit immunised with Percoll-purified merozoites originating from a panel of *B. divergens *strains, in order to highlight as many parasitic proteins as possible. To pinpoint the proteins involved in the invasion process (i.e. those potentially secreted and thus in contact with the immune system during natural infection), we chose to use sera from two calves experimentally infected with *B. divergens *strains C139 and IV35 respectively [34] and from four sheep experimentally infected with *B. divergens *strain Rouen 87 [35]. To avoid the production of antibodies against RBC proteins, the calves and sheep were infected with parasites cultivated in vitro in their own erythrocytes.

### Analysis of proteins contained in the invasion test supernatant (SDS-PAGE electrophoresis and Western blotting)

The conditions of efficient invasion were determined from the above assays. In summary, the merozoites were prepared by high-voltage electroporation, then incubated with 5% RBC in RPMI 1640 + 1 mM CaCl2 for 10 min. After incubation, the culture was centrifuged (16 000 g, 10 min, 4°C) to eliminate the RBC and remaining free parasites. After addition of Protease Inhibitor Mix 1:100 (Amersham Bioscience), the supernatant was concentrated 12 times by Centrifugal Filter Devices (Millipore-Amicon ultra-15, Billerica, USA) (10 000 *g*, 2 h, 4°C) with a 10 kDa cut-off. Potential contamination with erythrocyte proteins derived from the preparation of parasites for invasion was determined by electroporating non-infected bovine erythrocytes under the same conditions. The resulting pellet was added to the invasion medium, then the supernatant was collected and concentrated in the same way as described above.

The concentrated supernatants were separated on a 12% SDS-PAGE gel, stained with Coomassie Brilliant Blue R-250 or transferred to a nitrocellulose membrane, which was blocked with 10% skimmed milk in TBS/0.1% Tween 20 (TBSt) overnight. After each incubation, all washes were done three times for 5 min in TBSt. The blots were incubated for 1 h in sera from immunised rabbits, or experimentally-infected calves or sheep, diluted 1/100 in TBSt with 5% skimmed milk. After washing, the membranes were incubated with anti-IgG (anti-rabbit IgG; anti-bovine IgG or anti-sheep IgG coupled to alkaline phosphatase, Sigma Aldrich, Milwaukee, USA) diluted 1/1000 in TBSt with 5% skimmed milk for 30 min. The colorimetric reaction was carried out for 5 min with BCIP/NBT liquide substrate system (Sigma Aldrich).

### Inhibition of invasion with anti-sera

One calf serum (C139) and one sheep serum (S-M-SC) were then selected on the basis of the WB analysis and used to validate our protocol in an invasion inhibition assay and to determine the potential function of the proteins detected during invasion. Different dilutions (1/2, 1/4, 1/8) of the antisera were added separately to the invasion medium, before the addition of live merozoites prepared by electroporation. Their effects on invasion were compared to those of the same dilutions of sera from non-infected calves or sheep. After incubation for 10 min, the RBC were washed in RPMI 1640, then re-incubated in culture medium for 5 h before parasitemia control.

### Statistical analysis

A non-parametric test was used for the statistical analysis. Wilcoxon was used for the comparison between two elements, and Kruskal Wallis was used for comparison of more than two elements. The test was considered "statistically significant" when the p-value was less than 0.05.

## Results

### Percoll separation allows elimination of the extra-erythrocytic parasites and the calculation of invasion efficiency

**Figure 1:**
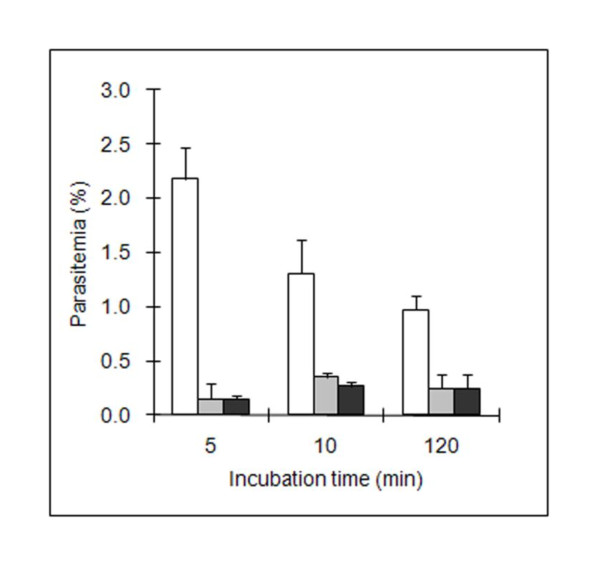
**Improvement of invasion efficiency evaluation**. Merozoites collected from culture supernatant were used to perform the invasion assay in RPMI 1640. Parasitemia was counted before (white bars), after Percoll separation (grey bars) and after 5 h incubation in culture medium (black bars). Each figure represents the average value of the triplicate assays and error bars indicate the standard deviation.

Parasitemia was measured before and after Percoll separation, and after 5 h incubation in culture medium to determine the efficiency of Percoll separation in eliminating parasites that did not invade erythrocytes during the invasion test and to control the non-toxicity of Percoll on intra-erythrocytic parasites. These measurements were performed 5, 10 and 120 min after the initial contact between RBC and merozoites. Regardless of the time of contact, a significant decrease in parasitemia from 14 fold (5 min) to 4 fold (120 min), was obtained after Percoll separation (Figure 1). The observed decrease in parasitemia according to the time of contact before Percoll separation was not correlated with a corresponding increase of invading parasites (after Percoll). No significant differences in parasitemia were observed before or after additional incubation in culture medium at any of the three contact times (Figure 1). That is to say that Percoll is compatible with the subsequent in vitro growth of *B. divergens*.

### Erythrocyte invasion is more efficient in RPMI 1640 supplemented with Ca^2+^

**Figure 2:**
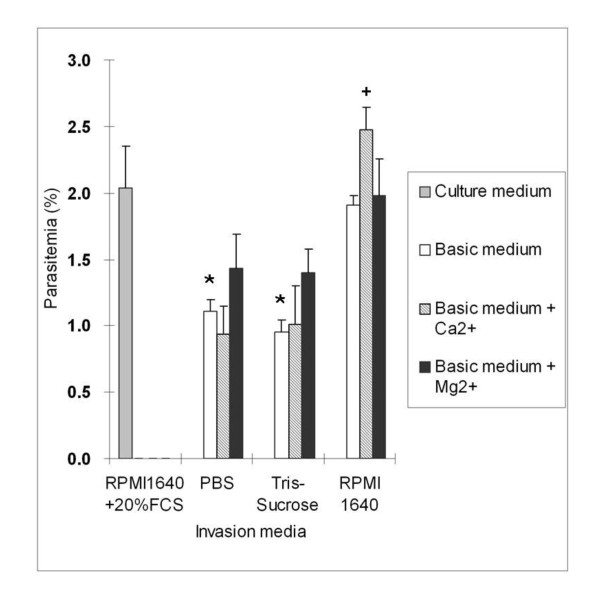
**Influence of medium composition on erythrocyte invasion efficiency**. The invasion assays were performed in 3 basic media (white bars), and compared to the complete medium (gray bar). CaCl_2 _(striped bars) or MgCl_2 _(black bars) were added at a concentration of 1 mM into the basic media separately. Each figure represents the average value of parasitemia from the triplicate assays and error bars indicate the standard deviation. Significant differences (*P *< 0.05) between complete medium and basic media are indicated with stars, while significant differences due to addition of calcium of magnesium in each basic medium are indicated with crosses.

In order to choose a suitable synthetic medium for performing the invasion test, we compared the invasion efficiency in culture medium (RPMI 1640 + 20% FCS) with the invasion efficiencies obtained using three synthetic media (PBS, Tris-Sucrose and RPMI 1640). Calcium and magnesium were added to each synthetic medium to find out if they could improve invasion efficiency. Free parasites were obtained by RBC osmotic lysis, and a 30 min incubation time was used. Among the three synthetic media tested, the same invasion efficiency as the culture medium was only obtained with RPMI 1640 (Figure 2). Parasitemia was significantly lower than the control when PBS or Tris-Sucrose was used. Adding calcium or magnesium had no significant effect except for RPMI 1640 supplemented with calcium. Therefore RPMI 1640 + 1 mM CaCl_2 _was selected for the in vitro invasion assays.

### Free, viable, and invasion-competent parasites were obtained by high-voltage electroporation

**Figure 3:**
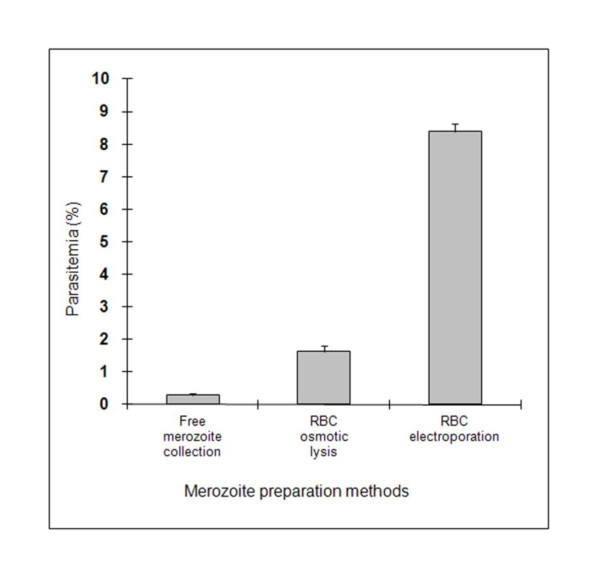
**Influence of the methods of merozoite preparation on erythrocyte invasion efficiency**. Parasites from the culture medium were collected by three different methods. The invasion assays were performed in RPMI 1640 + 1 mM CaCl_2_. The experiment was performed twice with similar results, and one of them is presented here. Each figure represents the average value of the triplicate assays and error bars indicate the standard deviation.

Starting with the same culture material, RBC osmotic lysis and RBC electroporation produced 5 times and 29 times more "invasion-competent" parasites respectively than merozoites from the culture supernatant (Figure 3). Similar results were obtained in a second assay (data not shown).

### *B. divergens *invasion of the erythrocyte is a rapid process

**Figure 4:**
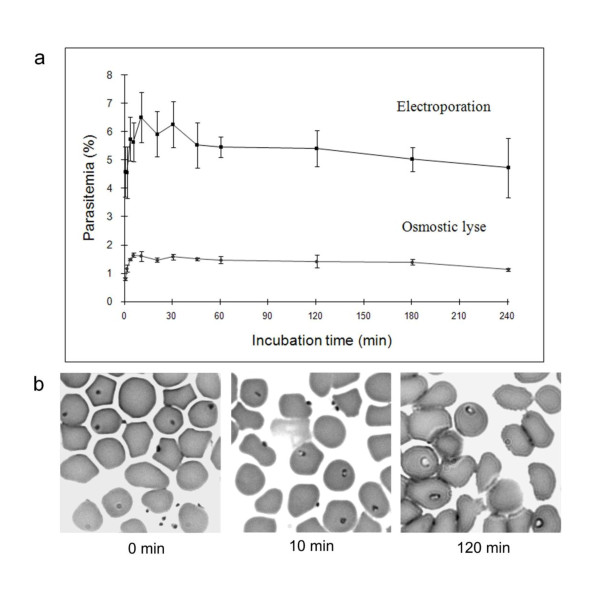
**a). Kinetics of erythrocyte invasion. Curves indicate the percentage of erythrocytes invaded with parasites prepared by high-voltage electroporation (squares) or by osmotic lysis (rounds) according to the time of incubation**. Each figure represents the average value of parasitemia from the triplicate assays and error bars indicate the standard deviation. b). Photomicrographs of bovine erythrocytes following an invasion assay with merozoites prepared by electroporation. The photos were taken immediately after contact and at 10 min and 120 min.

*B. divergens *parasites prepared by RBC lysis (osmotic and electroporation) were used to follow the kinetics of invasion by analysing parasitemia after contact with RBC ranging from 0 to 240 min. Although a huge difference in the maximum parasitemia attained in these two assays was observed (which confirmed the choice of electroporation as the better method of parasite preparation), the trends of the two curves were comparable (Figure 4a). *B. divergens *invasion of RBC occurred within seconds. A contact of 45 s (the time required to mix RBC with parasites, and to perform Percoll separation) was indeed sufficient to attain 50% (RBC osmotic lysis) and 70% (RBC electroporation) of maximum parasitemia. Ten minutes after the initial contact, all "invasion-competent" parasites had penetrated the RBC, since no further increase in parasitemia was observed over time. Microphotographs of Giemsa-stained smears prepared immediately after contact (about 5 s), at 10 min and 120 min after contact and before Percoll separation were taken to show the changes in parasite morphology (Figure 4b). Free parasites, some adhering to the RBC surface, were visible after contact (0, 10 min). Their quantities seemed to diminish with time (120 min). Intra-cellular parasites were visible immediately after the contact with RBC. Their shape changed rapidly, from condensed intra-erythrocytic black particles at the very beginning, to trophozoites with the appearance of cytoplasms (white areas) inside the parasite at ten minutes, which expanded to become clearly visible at 120 min. Parasites with homogeneous size and shape were still visible 120 min after the beginning of the culture.

### The intra-erythrocytic development of *B. divergens *is asynchronous

The development of *B. divergens *after invasion was followed by calculating at different incubation times the percentage of parasites at the following developmental stages: trophozoite, beginning and end of cellular division (Table 1). To improve synchronisation, late invasions were avoided by eliminating extra-erythrocytic parasites 10 min after the contact between RBC and parasites. Up to 3 h after RBC invasion, the only parasite forms observed in the culture were trophozoites (Table 1). The onset of division was observed at 4 h. The proportion of the population at this stage increased for at least 2 h, peaked around 6 h after invasion and then decreased. Fully divided parasites were observed 1 h after the onset of division, and accounted for about one third of the observed parasite population 9 h after invasion. The trophozoite population exhibited a considerable decline after invasion, from 6 h on, but remained the main population detected. Eight hours after invasion, about 30% of the parasites (approximate sum of parasite percentages seen at the end of division from 5 to 8 h) had fully divided to produce about 8 × 10^7 ^merozoites/mL, each invading a new RBC and resulting in a new trophozoite.

**Table 1:** Percentage of *B. divergens *parasites at the stages of trophozoite, onset of division and end of cellular division following an invasion assay.

	Trophozoite (%)	Onset of division (%)	End of division (%)
3 h	100	0	0

4 h	98.7	1.3	0

5 h	96.6	1.5	1.9

6 h	88.8	6.0	5.2

8 h	75.7	2.6	21.7

9 h	65.6	1.9	32.5

Parasites at each stages were counted from 3 to 9 h after the contact between free parasites and red blood cells.

### RBC can be invaded by two *B. divergens *merozoites

**Figure 5:**
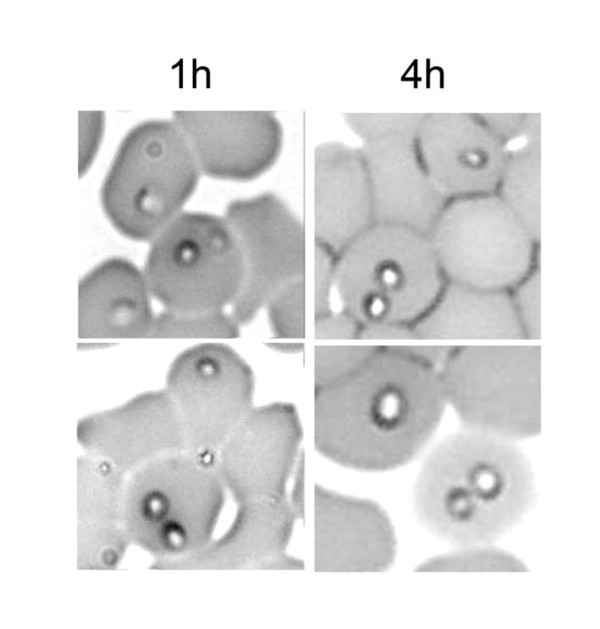
**Photomicrographs demonstrating the presence of two trophozoites in one RBC 1 h and 4 h after the start of invasion**.

Occasionally, after an invasion assay, two trophozoites were observed within the same RBC (Figure 5) and accounted for about 3% of the infected RBC. Their occurrence was observed just after invasion. They were similar in shape and size not only within a double infected erythrocyte, but also compared to single troprozoites, and this was observed 1 h and 4 h after invasion (Figure 5). Their presence in such an assay indicates the ability of two merozoites to invade a single target cell.

### *B. divergens *antigens were released into the environment during the invasion assay

**Figure 6:**
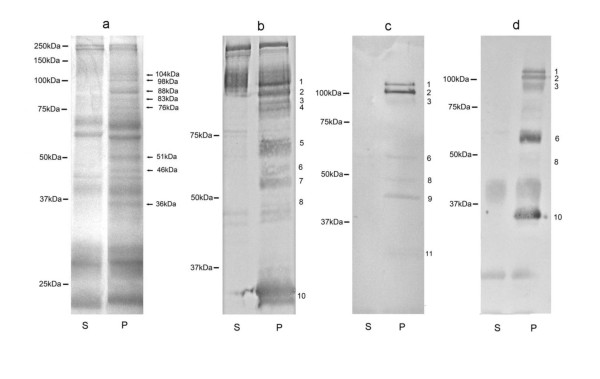
**a). SDS-PAGE of proteins released into the supernatant during erythrocyte invasion**. b, c, d). Proteins recognised by serum of rabbit, representative calf C139 and sheep S-M-SC respectively by Western blotting. Lane S, supernatant of invasion assay without parasites. Lane P, supernatant of invasion assay with parasites. The bars on the left of each photograph indicate the molecular masses of standard markers. Arrows indicate *B. divergens*-derived proteins. Band Nos. 1 to 11 are antigens which might be involved in the invasion process.

The proteins collected in the supernatant were analysed by SDS-PAGE. As a result of RBC lysis, many contaminating RBC proteins were present in the control lane (lane S) as well as in the test lane (lane P) (Figure 6a). The main parasite-derived proteins collected during the invasion assay (molecular weights ranging from 36 kDa to 104 kDa) are highlighted with arrows on lane P.

The collected proteins were also analysed by Western blotting with different *B. divergens *antisera. Many contaminating RBC proteins were recognised by rabbit antiserum, thereby confirming the presence of RBC contamination in the parasite preparation used for immunisation (Figure 6b). As expected, few (Figure 6d) or none (Figure 6c) of these proteins were detected in sera obtained from experimentally-infected animals. Three proteins with molecular weights of about 110 kDa, 102 kDa, and 95 kDa (respectively No. 1, 2, 3 on Figure 4) were recognised by all tested sera (7 sera in total, 3 of them shown as representative), even by calf sera collected at an early stage of infection (14 days) (Figure 6c). The other antigens were detected in different combinations depending on the serum.

### Antisera from experimentally-infected animals inhibit *B. divergens *invasion

**Figure 7:**
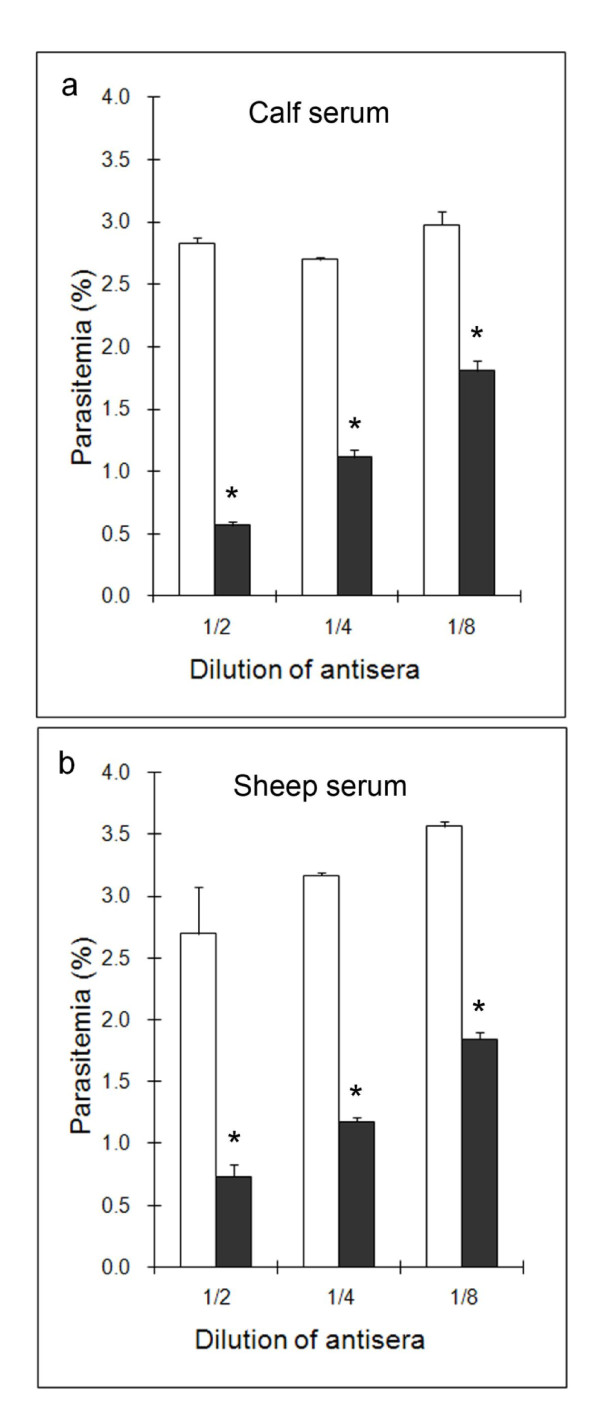
**Inhibition of invasion by sera directed against *B. divergens***. Invasion was performed with antisera of calf C139 and sheep S-M-SC (black bars) and compared to the negative sera of calf and sheep (white bars). Each figure represents the average value of the triplicate assays and error bars indicate the standard deviation. Significant differences (*P *< 0.05) between antisera and negative sera are indicated with stars.

The invasion assay was used to test the inhibitory effect of two selected sera on invasion (Figure 7). For both sera, an inhibition of invasion was observed at each dilution tested (up to 1/8) and the effect was dose-dependent. Invasion was reduced by up to 80% in the case of calf antiserum. Sheep negative serum also had an inhibitory effect on invasion, an effect that had already been noted on growth of *B. divergens *[36]. It could therefore be postulated that the proteins detected by Western blotting with these sera had a role in the invasion process.

## Discussion

Studies of the invasion process in *Babesia *are of pivotal importance since only this Apicomplexan genus has infectious forms (merozoites and sporozoites) which start their host invasion directly by erythrocyte penetration. However, very little information is available on this process compared, for example, to that of *Plasmodium*. We focussed our studies on *B. divergens *because it is the major hemoparasite of cattle in Europe with zoonotic transmission. We developed an in vitro invasion assay to acquire knowledge of the process and molecules involved in invasion of the RBC by *B. divergens*.

The choice of medium used to obtain efficient invasion in vitro is crucial. Of the three media tested, RPMI 1640 + 20% FCS and RPMI 1640 exhibited the same invasion efficiency. Although serum is known to be essential for *Babesia *growth in vitro, we demonstrate here, as reported for *B. bovis *and *P. falciparum *[14,32], that it is not necessary for erythrocyte invasion by *B. divergens*. The invasion efficiency measured with PBS (used for the *B. bovis *invasion test in Franssen et al., [32]) and Tris-Sucrose was significantly lower. The relative invasion efficiencies with PBS compared to the control medium were comparable for *B. bovis *(about 60%) and *B. divergens *(54%). However, the positive effect of calcium addition to PBS was significant only in the case of *B. bovis *invasion. A positive and significant effect of calcium addition was observed in our study only when RPMI 1640 was used (parasitemia increased by 30%). The role of extra-cellular calcium in merozoite invasion of the erythrocyte is well documented for *P. falciparum *[37] and *B. bovis *[32]. In our case, its effect depended on the medium.

Compared to the two other methods of merozoite preparation (collection of free merozoites in the culture supernatant and liberation of intra-cellular merozoites by osmotic lysis), electroporation of infected RBC was able to provide a greater number of "invasion-competent" parasites. It is extremely difficult to estimate the number of free and alive parasites due to their size and aggregation. Moreover, parasites might remain in the erythrocyte ghosts after lysis and be unable to invade a new RBC. The best way to compare the quality and quantity of "invasion-competent" parasites is to perform an in vitro invasion assay. Thus, a Percoll separation and reincubation in culture medium were performed to guarantee the reliability of the estimated invasion efficiency. Due to the death of extra-erythrocytic parasites, a large number of them were actually free or adhering to the surface of RBC but not internalized, and were therefore efficiently removed by the Percoll separation. Indeed, the parasitemia count obtained after incubation in culture medium is more reliable due to the size of the parasite.

Electroporation is a simple and effective method for the disruption of cell membranes by application of an external electric field. We confirmed the suitability of this method for *B. divergens*, as already described for *B. bovis *[32]. The amount of "invasion-competent" merozoites produced is large enough to establish a culture with parasitemia ranging from 5 to 14%, a range of parasitemia occurring during the acute phases of bovine Babesiosis. Such high parasitemia could not be achieved by osmotic lysis which repeatedly produced subsequent cultures with parasitemia ranging between 1 and 2%. The harmful effect of the medium used for RBC lysis was probably responsible for the death of the released parasites. A maximum parasitemia of 0.5% was obtained when free merozoites collected from the culture supernatant were used. This probably reflects the limited number of free merozoites in the supernatant or their low viability. According to our data, we collected about 5 × 10^6 ^"invasion-competent" merozoites from 40 mL of culture, calculated from the parasitemia after invasion (Figure 1). Other authors were able to collect about 10^8 ^merozoites from the same volume of culture [25]. There are two possible explanations for this difference. Firstly, the maximum parasitemia values of the initial cultures were different (20% vs. 70%, due to the erythrocyte type, bovine vs. human), which would influence the amount of free merozoites. Secondly, only about 10% of the collected merozoites were demonstrated to be "invasion-competent" (Figure 1), a characteristic that was not investigated by the authors. As demonstrated in our study, *B. divergens *invasion is an extremely rapid process meaning that "invasion-competent" merozoites remain freely in the medium for a very short time. The merozoites collected from the supernatant were probably those that had remained for a long time in the medium and were thus probably "non-invasion-competent" (dead or non-viable).

By electroporation, 6.4 × 10^9 ^infected RBC were necessary to obtain a culture with 6% parasitemia. This corresponds to 4.8 × 10^7 ^infected RBC after invasion, that is to say that only 0.75% of the prepared parasites were "invasion-competent", i.e. viable merozoites. This low percentage might be explained by the asynchronous status of the culture. The invasion yield could be optimized by using a synchronized culture to increase the proportion of geminate-stage parasites.

The in vitro invasion assay presented in this study permits the analysis of the kinetics of invasion, especially its rapidity. As observed for other Apicomplexan parasites, the invasion of *B. divergens *proceeded rapidly after the initial contact between parasites and RBC, since 70% of the "invasion-competent" parasites invaded a RBC within 45 s, and the maxium level of invasion was attained within 10 min. This result corresponded to the observation of *B. bovis *erythrocyte invasion that the attachment to RBC was achieved within 5 min, whereas 41% of the maximum level of invasion was attained after 15 min, and the maximum after 1 h [32]. During the asexual multiplication of intra-erythrocytic Apicomplexans, the merozoite is the only stage in contact with the host immune system. *B. divergens *and other Apicomplexan parasites have developed an efficient invasion mechanism to minimise the time of contact and ensure survival in their vertebrate hosts.

Another application of this in vitro invasion assay is the collection of proteins released by the parasites during the invasion process. Host-cell invasion by apicomplexan parasites involves the successive exocytosis of secretory organelles into the RBC and environment [38]. These proteins, present in in vitro culture, can induce a high degree of protection against virulent heterologous challenge exposure [39,40]. They are thought to be potential sources of antigens to induce protective immunity. Moreover, their characterisation should provide a better understanding of the molecular support underlying host cell invasion.

A minimum invasion level has to be reached to ensure that the amount of proteins is large enough for their characterisation, which is why an efficient method of merozoite preparation is crucial to the invasion assay. With our assay we were able to collect, in 1 mL of invasion medium, the proteins secreted by 4.8 × 10^7 ^to 1.1 × 10^8 ^invasion competent parasites within 10 min, depending on the assay (with parasitemia of subsequent cultures ranging from 6 to 14%).

Proteins collected from the invasion assay supernatant were labelled by Western blotting with different antisera. As expected, the antibodies of rabbit antisera prepared by immunisation with Percoll-separated merozoites recognised numerous proteins including probably house-keeping ones. On the contrary, sera from experimentally-infected animals (calves or sheep) recognised fewer proteins (about 6). These sera are most probably enriched in antibodies against the most antigenic proteins, and among them, merozoite surface antigens or proteins secreted during invasion by the apical organelles of the merozoite (rhoptries and micronemes) could be included. Among these proteins, some were recognised by calf sera collected early during the infection process (proteins No.1, 2, 3, Figure 6c). Others (proteins No. 8 and 9, Figure 6c) could correspond to already described invasion-related proteins, such as AMA-1 (~48 kDa, after the precursor cleavages) [25] and RAP-1 (~46 kDa) [29]. An invasion inhibition assay was performed to determine the participation of these recognised proteins in the invasion process. The significant and dose-dependent inhibitory effect of sera from infected animals on erythrocyte invasion suggests such a role. Further investigations are needed for their identification as potential vaccine candidates.

The asexual multiplication of *B. divergens*, in vivo as well as in vitro, is asynchronous and methods of obtaining a synchronised *Babesia *culture are lacking. Research using synchronised culture is essential to study DNA transcription and protein expression at different parasitic stages. Synchronised cultures could also be used to identify the precise cellular steps (DNA replication, cell division) targeted by drugs. *B. bovis *in vitro invasion with isolated parasites was not able to provide a synchronised culture [32]. For the purpose of obtaining a synchronised culture of *B. divergens*, non-lysed RBC (infected or not) were eliminated after electroporation. Moreover, the invasion window time was restricted to 10 min, and could be further reduced by removing extra-erythrocytic parasites from the RBC earlier to obtain an improved synchronised start. Even so, the intra-erythrocytic development of the parasite did not remain synchronised. Parasites at the stage of onset of division were detected from 4 h on and still at 9 h after invasion included, thus indicating the extremely heterogeneous intra-cellular development of this parasite. This results in the mix of the trophozoites from the first generation and that of the second generation, which explained the maintenance of a trophozoite population in the culture, even when the time window for invasion to occur was limited to 10 min. However, this assay could be used to study gene transcription and expression in relation to the onset of main cell-cycle features, such as DNA replication or cell division events. Synchronisation is well developed for *Plasmodium *[41-45], *Toxoplasma *[46] and *Theileria *[47], but was barely achieved for *Babesia *[32]. A synchronised culture of *P. falciparum *with a large age range (3-5 h) was obtained by density gradients [41,42], differential osmotic lysis of parasitised erythrocytes [43], temperature shifts [44], and recently optimised to a specific cell-cycle phase with growth inhibitors [45]. Further improvement of the synchronisation of *B. divergens *cultures will probably rely on drugs that specifically block key points in the cell cycle. However, the effects of such molecules on *B. divergens *remain uncharacterised.

We established an efficient in vitro invasion assay for *B. divergens*. Thanks to this assay, we demonstrated that RBC invasion occurred in less than 45 s, and that the invasion of one erythrocyte by two merozoites is possible. Synchronisation, even partial, of a culture could allow transcription and translation studies during the cell-cycle. By uncoupling invasion and intra-erythrocytic development, studies of the actions of inhibitory compounds that specifically target these stages would be feasible. The presence in the assay medium of proteins involved in invasion is suspected, since inhibition of invasion with antibodies from experimentally-infected animals was obtained. The invasion assay provides the possibility of more accurate and specific comparisons of the invasion process between *B. divergens *and other Apicomplexa. More specifically, *Babesia*-specific characteristics such as the disappearance of the parasitophorous vacuole or more generally the molecular basis of host specificity could be investigated.

## Competing interests

The authors declare that they have no competing interests.

## Authors' contributions

YS carried out most of the experimental part with the help of LM for the invasion tests. YS and LM wrote the manuscript. AC was YS PhD supervisor and revised the manuscript. EM and LM participated in the design, coordination and revision of the study. All authors read and approved the final manuscript.
